# An Unexpected Cause of a Subcutaneous Nodule: A Case Report of *Dirofilaria* Infection

**DOI:** 10.1155/2012/191245

**Published:** 2012-01-15

**Authors:** Bruno Man-Hon Cheung, Yi-Lan Huang, Yie-Wen Lin, Yung-Sen Chang, Shian-Min Liu

**Affiliations:** ^1^Division of Infectious Diseases, Department of Internal Medicine, Tainan Municipal Hospital, No. 670, Chung Te Road, Tainan 701, Taiwan; ^2^Department of Surgery, Kaohsiung Municipal Gangshan Hospital, No. 12, Shou-Tian Road, Kaohsiung City 820, Taiwan; ^3^Office of Deputy Superintendent, Tainan Municipal Hospital, No. 670, Chung Te Road, Tainan 701, Taiwan; ^4^Department of Pediatrics, National Taiwan University Hospital, No. 7, Chung-Shan South Road, Taipei 100, Taiwan; ^5^Department of Pathology, Tainan Municipal Hospital, No. 670, Chung Te Road, Tainan 701, Taiwan

## Abstract

Humans are not natural hosts of *Dirofilaria*; however, pulmonary or subcutaneous infections may occur through mosquitoes transmission. Patients presenting with simple subcutaneous nodules may not seek early medical attention, and hence systemic involvement through hematogenous spread may occur. Definitive diagnosis of Dirofilaria infection is made by histopathological examinations of the infected tissues. We report a patient with an incidental diagnosis of Dirofilaria infection confirmed by histopathological findings of a subcutaneous nodule on the right thigh. The source of infection remains unknown.

## 1. Introduction

Dirofilariasis is caused by a zoonotic filarial nematode. It is transmitted to humans by *Culex*, *Aedes*, or *Anopheles* mosquitoes, which ingest blood-containing microfilaria from affected dogs, cats, or raccoons. The filariae, also known as the “heartworm”, lodge in the right ventricle and pulmonary arteries of hosts, causing dyspnea and anemia. It is most commonly seen in tropical and subtropical areas, and canine infection is more common in southern Europe [1–3]. Human dirofilariasis is rare. It usually presents with nodular lesions in the lung, subcutaneous tissues, peritoneal cavity, or eyes. *Dirofilaria* does not mature into fully gravid worms in humans. Reported cases of Dirofilaria infection in humans included two species, *Dirofilaria immitis* and *Dirofilaria repens *[3–14] and all were diagnosed by histopathological examinations of specimen biopsied from the infected tissues.

## 2. Case Report

**Figure 1:**
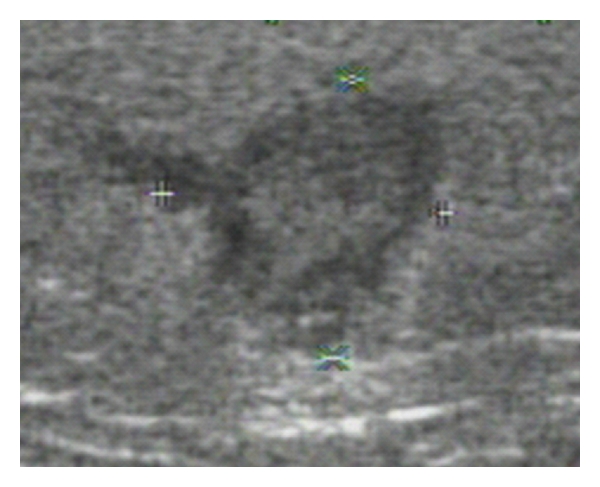
A subcutaneous nodule was detected by sonogram. Size: 10.3 × 10 mm. Depth: 11.3 mm.

**Figure 2:**
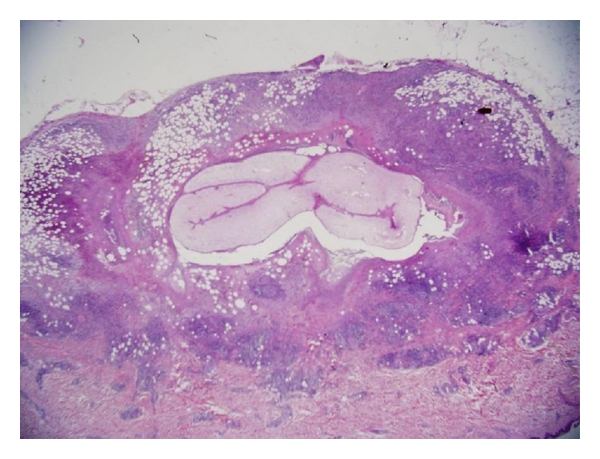
Microscopic view of cross-sections of the nematode (hematoxylin and eosin stain, original magnification 125x).

**Figure 3:**
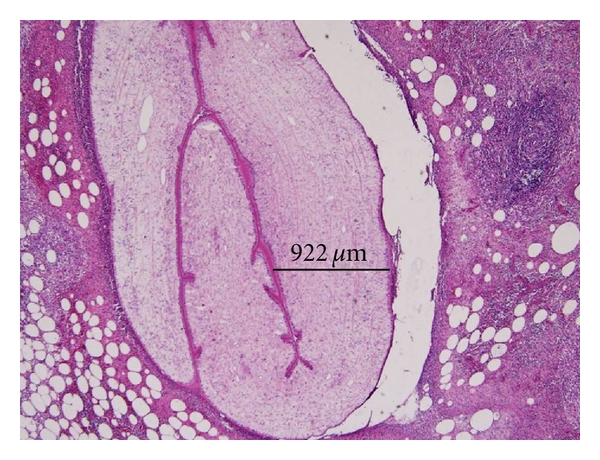
Microscopic view of cross-sections of the nematode (hematoxylin and eosin stain, original magnification 400x).

A 46-year-old, previously healthy women presented with a subcutaneous nodule at her right thigh for 4 months. The nodule enlarged gradually. Physical examination showed a well-defined, elastic, nontender, immovable, and oval-shaped nodule about 2 centimeters in diameter at the medial side of her right thigh. Initial impression was an atheroma or subcutaneous cyst. Soft tissue sonography showed that the nodule was adherent to the epithelia (Figure 1). Although the clinical characteristics of this nodule implied that it is benign in nature, excision of the nodule was performed due to its gradually enlarging size. An encapsulated nodule around 2.2 cm in diameter was resected. Unexpectedly, pathological examination revealed Dirofilaria infection (Figures 2 and 3). Chest X-ray showed no abnormal pulmonary nodules. Hemogram found no eosinophilia. As for the contact history, the patient has a 6-month-old pet dog, but has no contributory travel or contact history. We did blood tests on the pet dog for two times, but there was no evidence of dirofilariasis. At followup, the patient had no recurrence or newly onset nodular lesions in the skin or the lungs.

## 3. Discussion

Most human cases of dirofilariasis have been reported in America, Japan, and India [5–9, 11, 14]. In Taiwan, Huang et al. reported a case of dirofilariasis presenting with a subcutaneous nodule [15], and Tsung and Liu reported a case with a pulmonary nodule [16]. According to Wu's report, the estimated incidence of canine dirofilariasis in dogs older than 2 years of age is around 25% in Taiwan, which is higher than that of 10 years ago [17]. The highest incidence rate is in Nantou County in mid-Taiwan, up to 41% [17]. Lai et al. found that in mid-Taiwan, *Aedes albopictus* is more efficient at transmitting dirofilariae than *Culex quinquefasciatus*, but *Culex quinquefasciatus* is still the main source of transmission [18]. Our patient resides in Kaohsiung City of southern Taiwan and had no travel history. Her pet dog was only 6 months old and had no evidence of Dirofilaria infection. Therefore, the exact source of infection remains unknown in this patient.

Patients seldom seek medical help for a subcutaneous nodule other than for cosmetic concerns [3, 14]. Most subcutaneous nodules are benign [19]. Unless the nodules are situated at lymph nodes or are adjacent to major vessels such as the femoral arteries and veins, or major vessels of the neck, sonography is not routinely performed before surgical excision. By sonography, distinction between subcutaneous lipomas and epidermal cyst can be made. Subcutaneous lipomas are usually separated from the epidermis by a layer of subcutaneous tissue, while epidermal cysts usually are directly adjacent to the epidermis. The sonographic image of our patient resembled an atheroma [19] rather than a subcutaneous lipoma. However, the exact composition of the nodule could not be clearly identified solely by sonography. Histopathological examination remains the gold standard to confirm the diagnosis.

Human Dirofilaria infection, whether presenting as subcutaneous nodules, pulmonary nodules, pelvic cavity nodules, or nodules in the eye, must be diagnosed by histopathological examinations of the infected tissues. Eosinophilia is found occasionally [3]. For lung or pelvic cavity nodules, extensive survey is usually required to differentiate other diseases such as eosinophilic granuloma (histiocytosis X), non-Hodgkin's lymphoma, sarcoidosis, tuberculosis, Wegener' granulomatosis, primary malignant tumors, echinococcosis, histoplasmosis, hookworm infection, or Ascariasis [20]. Fortunately, our patient presented with a solitary subcutaneous nodule without eosinophilia or other organ involvement. There was no evidence of disease recurrence after complete resection of the nodule.

## 4. Conclusions

Dirofilariae are transmitted to humans via mosquitoes and may lodge in the lung, subcutaneous tissue, pelvic cavities, or eyes. It has been proven that canine dirofilariasis and mosquitoes capable of Dirofilaria transmission do exist in Taiwan. Experience from this case suggests that dirofilariasis should be considered as a differential diagnosis in patients presenting with subcutaneous nodular lesions, especially in those with relevant travel or contact history suggestive of Dirofilaria infection.

## References

[1] Vakalis NC, Himonas CA (1997). Human and canine dirofilariasis in Greece. *Parassitologia*.

[2] Orihel TC, Eberhard ML (1998). Zoonotic filariasis. *Clinical Microbiology Reviews*.

[3] Permi HS, Veena S, Kishan Prasad HL, Kumar YS, Mohan R, Shetty KJ (2011). Subcutaneous human dirofilariasis due to Dirofilaria repens: report of two cases. *Journal of Global Infectious Diseases*.

[4] Font RL, Neafie RC, Perry HD (1980). Subcutaneous dirofilariasis of the eyelid and ocular adnexa. Report of six cases. *Archives of Ophthalmology*.

[5] Asimacopoulos PJ, Katras A, Christie B (1992). Pulmonary dirofilariasis; The largest single-hospital experience. *Chest*.

[6] Pampiglione S, Canestri Trotti G, Rivasi F (1995). Human dirofilariasis due to Dirofilaria (Nochtiella) repens: a review of world literature. *Parassitologia*.

[7] Shah MK (1999). Human pulmonary dirofilariasis: review of the literature. *Southern Medical Journal*.

[8] Skidmore PJ, Dooley DP, Dewitt C (2000). Human extrapulmonary dirofilariasis in Texas. *Southern Medical Journal*.

[9] Gautam V, Rustagi IM, Singh S, Arora DR (2002). Subconjunctival infection with Dirofilaria repens. *Japanese Journal of Infectious Diseases*.

[10] Maltezos ES, Sivridis EL, Giatromanolaki AN, Simopoulos CE (2002). Human subcutaneous dirofilariasis: a report of three cases manifesting as breast or axillary nodules. *Scottish Medical Journal*.

[11] Padmaja P, Kanagalakshmi, Samuel R, Kuruvilla PJ, Mathai E (2005). Subcutaneous dirofilariasis in southern India: a case report. *Annals of Tropical Medicine and Parasitology*.

[12] Kramer LH, Kartashev VV, Grandi G (2007). Human subcutaneous dirofilariasis, Russia. *Emerging Infectious Diseases*.

[13] Abdel-Rahman SM, Mahmoud AE, Galal LAA, Gustinelli A, Pampiglione S (2008). Three new cases of human infection with Dirofilaria repens, one pulmonary and two subcutaneous, in the Egyptian governorate of Assiut. *Annals of Tropical Medicine and Parasitology*.

[14] Khurana S, Singh G, Bhatti HS, Malla N (2010). Human subcutaneous dirofilariasis in India: A report of three cases with brief review of literature. *Indian Journal of Medical Microbiology*.

[15] Huang SL, Liu YH, Chen W (2006). Subcutaneous dirofilariasis caused by Dirofilaria immitis mimicking a large epidermal cyst. *Journal of the European Academy of Dermatology and Venereology*.

[16] Tsung SH, Liu CC (2003). Human pulmonary dirofilariasis in Taiwan. *Journal of the Formosan Medical Association*.

[17] Wu CC, Fan PC (2003). Prevalence of canine dirofilariasis in Taiwan. *Journal of Helminthology*.

[18] Lai CH, Tung KC, Ooi HK, Wang JS (2001). Susceptibility of mosquitoes in central Taiwan to natural infections of Dirofilaria immitis. *Medical and Veterinary Entomology*.

[19] McCarthy (1990). *Plastic Surgery, Vol. 6*.

[20] Elder D, Elenitsas R, Jaworsky C, Johnson B (1997). *Lever’s Histopathology of the Skin*.

